# Multi-Scale Histogram-Based Probabilistic Deep Neural Network for Super-Resolution 3D LiDAR Imaging

**DOI:** 10.3390/s23010420

**Published:** 2022-12-30

**Authors:** Miao Sun, Shenglong Zhuo, Patrick Yin Chiang

**Affiliations:** State Key Laboratory of ASIC and System, Fudan University, No. 825, Zhangheng Road, Shanghai 201203, China

**Keywords:** SPAD sensor, LiDAR imaging, neural network, super resolution

## Abstract

LiDAR (Light Detection and Ranging) imaging based on SPAD (Single-Photon Avalanche Diode) technology suffers from severe area penalty for large on-chip histogram peak detection circuits required by the high precision of measured depth values. In this work, a probabilistic estimation-based super-resolution neural network for SPAD imaging that firstly uses temporal multi-scale histograms as inputs is proposed. To reduce the area and cost of on-chip histogram computation, only part of the histogram hardware for calculating the reflected photons is implemented on a chip. On account of the distribution rule of returned photons, a probabilistic encoder as a part of the network is first proposed to solve the depth estimation problem of SPADs. By jointly using this neural network with a super-resolution network, 16× up-sampling depth estimation is realized using 32 × 32 multi-scale histogram outputs. Finally, the effectiveness of this neural network was verified in the laboratory with a 32 × 32 SPAD sensor system.

## 1. Introduction

LiDAR plays a vital role in the perception and localization of autonomous vehicles, but the depth information from raw sensors suffers from spatial and temporal noise and resolution in practical scenarios. Recently, dToF (direct Time of Flight)-based depth sensing emerged, but its pixel resolution and power consumption are hard to balance for the sensor designer. With super-resolution neural networks being more commonly used, depth maps can be up-sampled from low to high resolution with the guidance of RGB images. This enables dToF sensors to output high-quality depth maps within limited pixel number and power consumption. Some up-scaling works on depth maps that employ hardware and algorithms are introduced independently below.

In the field of sensor design, research about increasing the pixel density of SPAD sensors has been going on for decades, from the 128 × 128 sensor [1] to the MEGA pixel array based on the 3D-stacked process [2]. Due to the maturation of SPAD device technology, smaller numbers of pixels, higher fill factors, and lower dark count rates (DCRs) have been achieved [3]. Other than device improvements, lots of research has focused on digital signal processing. In [4,5,6], the chip area is dominated by the memory resource of histogram data storage, the Time-to-Digital Converter (TDC), and the depth calculation units. In addition, the speed of hardware is limited by the high-bandwidth IO requirements due to the huge amount of raw histogram data outputs. In [7], Zhang et al. develop a partial histogram method to save the digital logic and on-chip SRAM area in a ratio of 14.9 to 1. However, these digital signal processors only focus on hardware optimization. With limited flexibility, the trade-off between sensor performance and on-chip resource is difficult to achieve.

Recently, neural networks have been introduced to improve the quality of 3D imaging. In [8,9,10,11,12], a neural network is capable of recovering the depth information from a flood of reflected photons with significant spatial and temporal noise, such as multi-path reflection and ambient light. In addition, thanks to the spatial and temporal correlation within the neural network, the RGB and intensity information can be fully utilized as prior knowledge to further improve the 3D images. In [8], by employing a sparse point cloud and gray-scale data as the inputs to the 3D convolution neural network, the temporal noise is filtered out, and the spatial density is increased. In order to achieve a better end-to-end super-resolution imaging quality, the authors of [9] take the optical phase plate pattern and its spatial pattern distribution function as the neural network’s prior knowledge, which combines optical design and a reconstruction neural network. The authors of [10] propose a two-step interpolation scheme for high-speed 3D sensing, and a high-quality imaging system with a frame rate of over 1 k-fps is demonstrated. A multi-feature neural network using the first depth map and the second depth map is designed to obtain the up-sampling depth in [11], and the feasibility of object segmentation on a small batch is proven in [12].

To fill the gap between efficient hardware usage and algorithm design, a new algorithm combining a novel data format and a two-step neural network is proposed. Firstly, a multi-scale histogram is adopted on hardware to save on-chip complexity and keep more information. Secondly, a probabilistic encoder and Super-Resolution (SR)-Net are proposed to recover and up-sample the depth map. The probabilistic encoder is responsible for extracting the depth information from histogram bins. Based on this kind of design, a probabilistic encoder-based neural network is first introduced for the SPAD denoising problem. Generated by the probabilistic encoder, the low-resolution depth image is up-scaled with SR-Net. To recover the high-resolution depth image, U-Net-based SR-Net conducts the 4× up-sampling of the output depth of the probabilistic encoder. Thirdly, by simulating the depth maps using an open depth dataset, the neural network is trained for specific hardware design under the same parameters set in the depth sensor, which guarantees performance in real tests. Finally, by testing the algorithm in the laboratory, we show that the proposed solution can recover the depth image from 32 × 32 multi-stage histogram bins, which demonstrates feasibility when implementing the network from training datasets to the real world. This work is organized as follows: The principle of the proposed neural network is described in Section 2. The simulation details of the generated dataset and training results are shown in Section 3. In Section 4, the sensor design, hardware architecture, and data acquisition are introduced. Section 5 compares our method with other advanced LiDAR systems. Furthermore, the discussion and conclusion are presented in Section 5 and Section 6.

## 2. Principle of Proposed Probabilistic Deep Neural Network

TCSPC (Time-Correlated Single-Photon Counting) is used to estimate distance information as a classical method. The on-chip memory footprint needed to construct the full histogram of returned photons is immense; therefore, multi-scale histograms represent an efficient way to extract peak values. However, there are many concerns about multi-scale histograms: (i) For the temporal information presented by returned photons, one surface per pixel is assumed, since the peak extraction can only find one peak, which may not be valid at object edges. (ii) For a few counted photons, the simple max selection criterion would be very noisy and would probably lead to sub-par results, since depth estimation works by selecting the time bin with the maximum photon count and then running another measurement acquisition that samples finer time bins within the initially selected bin at the coarser scale. Considering these cons of partial histograms, we propose a combination algorithm with classical multi-scale histograms for temporal depth information extraction and U-net-based neural network for spatial up-scaling and denoising.

**Figure 1:**
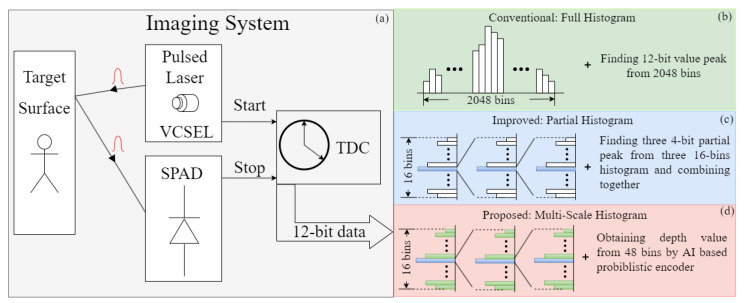
Comparison with previous works: (**a**) Imaging system including Vertical-Cavity Surface-Emitting Laser (VCSEL)-based TX, SPAD-based RX, and 12-bit TDC to calculate the output. (**b**) Conventional method, which costs 2048 bins and peak finding calculation. (**c**) Improved way to limit the bin number to 48 bins and use peak finding calculation for 3 histograms. (**d**) Method in this work, which takes 48 bins as the inputs and with which the depth value is obtained using the proposed AI neural network.

**Figure 2:**
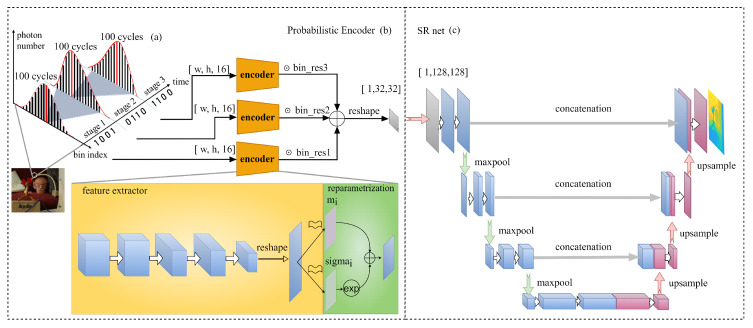
Architecture of the proposed neural network: (**a**) Sampling procedure of multi-scale histogram mode. In this experiment, in each stage, the VCSEL on hardware is driven 4 k times. According to the returned TDC results, the peak range is zoomed in the next stage. (**b**) Probabilistic encoder structure, which combines the CNN-based encoder part and the reparametrization trick. U-Net based SR-Net is shown in (**c**). SR-Net is responsible for the up-sampling task in this neural network.

As shown in Figure 1a, compared with the previous methods, a multi-scale histogram is adopted to save on-chip memory and calculation load for peak finding. The full histogram in Figure 1b is used at first to calculate the reflected photon number of each bin. Full histograms cost huge memory area and computation load for peak finding. Therefore, partial histograms are designed to save on-chip memory and calculation load. However, as shown in the partial histogram calculation in Figure 1c, all information except for the peak value is discarded during zooming into the next stage, which leads to inaccurate depth estimation. In the proposed multi-scale histogram method (Figure 1d), all 48 bins are taken as the inputs to the neural network. By training the neural network on the simulated dataset, an accurate depth map is obtained in this way. The architecture of the proposed neural network is demonstrated in Figure 2. Three 16-bin histogram distributions are obtained after 4 k pulses for each pixel. Due to the distribution of reflected photons following poison distribution P, the encoder part of the VAE [13] is introduced to learn the mean value (Equation (1)) for every independent distribution for every pixel. The reparametrization trick is adopted to obtain the estimation value of the Gaussian distribution with average $m_{i,j}$ and deviation $\sigma_{i,j}$.

(1) $$c_{i}=exp\left(\sigma_{i}\right)+m_{i}$$

The three histograms are constructed using different gratuities of the 12-bit TDC; therefore, the actual depth resolution ranges from TDC<11:8> to TDC<7:4>, to TDC<3:0>. Obtained with three multi-scale histogram probabilistic encoder branches, these initial multi-scale depth maps are multiplied by different ratios and added together as the input to the next up-sampling net. According to the experiments, the actual ratio is efficient and vital for the convergence of the neural network. The resolution of the TDC is used as prior knowledge for the multi-scale histogram. For the up-sampling net, the U-net [14] architecture is applied as SR-Net, which is capable of dealing with image segmentation and super-resolution issues well. Furthermore, its usage of multi-level concatenation contributes greatly to the confusion of multi-scale spatial information. Considering the hardware characteristics of SPAD imagers, this work aims to design an algorithm that avoids using other assistant information, such as RGB images, albedo maps, and intensity images. Although the RGB image contributes a lot to the final up-sampled image quality, it poses a challenge to the misalignment problem between RGB image pixels and SPAD array pixels. Compared with the RGB image, the density map (confidence map) is an alternative method that consumes fewer registers and obtains the confidence guidance map from a hardware point of view. Owing to the time consumption for collecting real SPAD measurements, the training datasets in this experiment were simulated using the Middlebury dataset [15] with 18 scenes. Compared with the other simulated datasets used in super-resolution tasks, multi-scale histograms are generated for each training image and test image. To mimic the multi-scale histogram behaviors, firstly, the SPAD measurement histograms with 16 bins are simulated with the bin size of 7680 ps. Secondly, the histogram range is zoomed into 480 ps, and the same number of histogram bins is maintained. Finally, each bin has a resolution of 30 ps in the last stage. To tolerate the different noise levels in real scenes, the Signal-to-Background Ratio (SBR) ranges from 0.05 to 5. The simulated data for training the neural network are used according to Equation (2).

(2) $$h_{m}\left[n\right]∼P\left(N\left(\eta \gamma \tau \left[n\right]+\eta \alpha +d\right)\right)$$

where *n* is the sample index from objects, η is the detection efficiency, and γ is the reflection factor that is related to distance and object materials. The number of arriving photons, τ, follows independent distribution for pixel (i,j). Ambient photons α result from background lights, such as a fluorescent lamp, sunlight, and other potential light sources. The hot noise of the SPAD also brings some erroneous counts (*d*). Additionally, the error between probabilistic encoder outputs and true distance is evaluated using L2 (denoted as Mean Squared Error; Equation (3)).

(3) $$L2=MSE(exp\left(\sigma_{i}\right)+m_{i}-d_{i})$$

Under real lab conditions, the raw depth value is computed using MLE (maximum likelihood estimation). The depth value is calculated with the full histogram in the lab, and $d_{i,j}$ is estimated using Equation (4) (cited from [10]).

(4) $$d_{i,j}=\frac{\sum_{t=max(1,d^{max}-1)}^{T,d^{max}+1}t*max\left(0,h_{i,j,t}-b_{i,j}\right)}{\sum_{t=max(1,d^{max}-1)}^{T,d^{max}+1}max\left(0,h_{i,j,t}-b_{i,j}\right)}$$

where *b* is the median of the bins used as the measured ambient level.

The entire neural network loss, *L* (Equation (5)), consists of the Root Mean Squared Error (RMSE) among the probabilistic encoder-predicted initial depth with 32 × 32 down-sampled depth value from the ground truth, the loss of the up-sampled depth image error and the 128 × 128 original depth value.

(5) $$L=\lambda *RMSE\left(x,\hat{x}\right)+\left(1-\lambda \right)*MSE\left(exp\left(\sigma_{i}\right)+m_{i}-d_{i}\right)$$

## 3. Simulation Setup and Experiments

**Figure 3:**
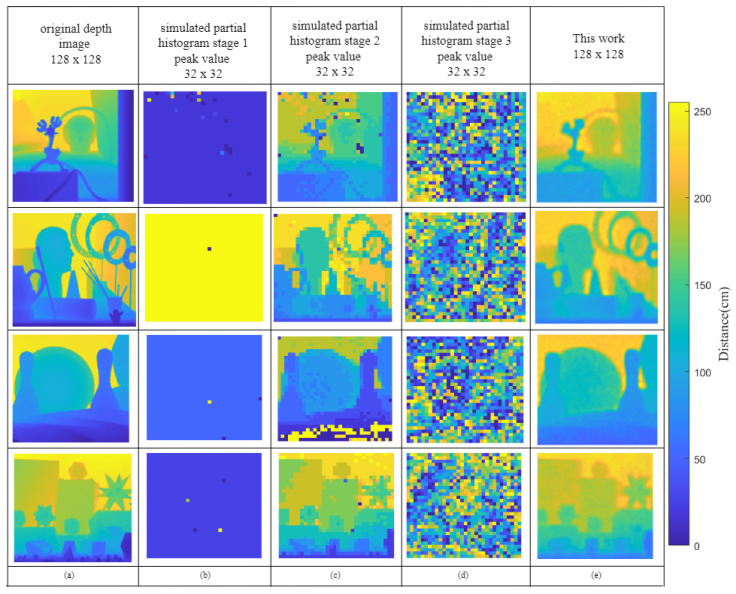
Simulated data used in this work based on the Middlebury dataset. (**a**) Original depth image used to generate the multi-scale histogram behavior inputs for the probabilistic encoder. (**b**–**d**) Peak values of three-stage outputs with SBR = 5. (**e**) Output results of this work.

Figure 3 demonstrates the simulated images of three multi-scale histograms using the test dataset with SBR = 5. Figure 3a shows one ground truth image from the test dataset. The processed peak value images for TDC<11:8>, TDC<7:4>, and TDC<3:0> are shown in columns (b), (c), and (d). The coarse range of TDC<11:8> showed the coarsest range of the detected objects with resolution of 7680 ps in this experiment, which means that the distance range in column (b) is above 1.115. In column (c), the data located in TDC<7:4> contain rich outline information of the objects; due to this, the greatest depth value in the simulated dataset is between 2 m and 3 m. Column (c) shows a distribution similar to the original object surface. In column (d), the least four significant bits of TDC<3:0> fill in more details, which show minor variations in the object surface. After 100 epochs of training, the neural network achieved a lower RMSE, with 0.022 m, on the test dataset.

## 4. Chip Description and Data Acquisition

**Figure 4:**
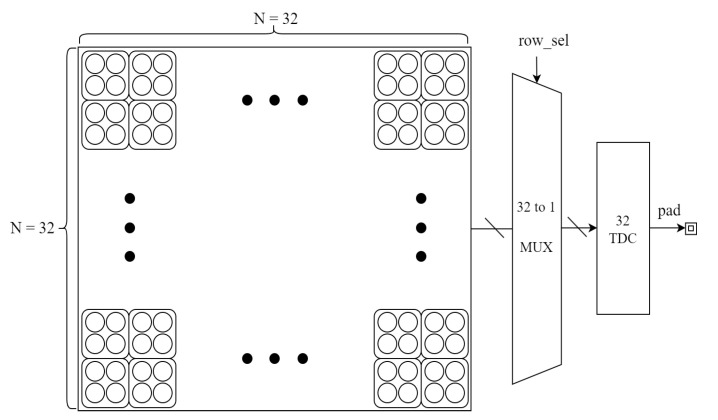
The block diagram of the tested chip with 32 line-shared TDCs and 32 × 32 macro SPAD arrays combined by 4 × 4 pixels.

The block diagram of the SPAD array and the TDCs is introduced in Figure 4. The sensor consisted of a 128 × 128 SPAD of 25 um in diameter and 32 shared TDCs. Measurements were performed with a dark count rate (DCR) of 100 kcps at 70 C, and photon detection probability (PDP) was $\tilde{1}\%$ at 940 nm. The low-resolution histogram was sampled by 4 × 4 combined pixels under a measured SNR condition of 14 signal photons and, on average, 450 noise instances in all pixels. Considering the affect that the coarse-grained TDC brings, the sampling numbers of three stages were configured as 2 k, 1 k, and 1 k. During the whole dynamic range of the TDC, the simulated probabilistic SNR for each detection was 14/450, and there were thousands of pulses to perform histogram calculation.

**Figure 5:**
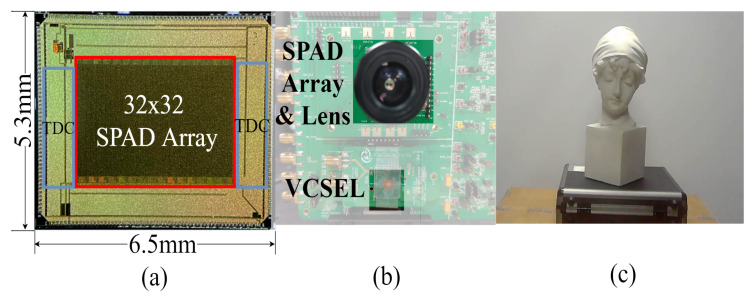
Experimental setup: (**a**) Layout graph of SPAD sensor and placement of vital circuits. (**b**) Evaluation board in this experiment, which was fabricated with on-chip multi-scale histogram circuit design. (**c**) Indoor target object.

Figure 5 shows the hardware platform including the SPAD array and VCSEL [16]. The SPAD array was fabricated with TSME 180 nm technology. The 32 × 32 SPAD array (Figure 5a) had an SPAD size of 128 × 128, and each 4 × 4 SPAD array was combined as an output to obtain the 32 × 32 depth map. The prototype of the imaging system is shown in Figure 5b, which was set parallel to the target statue 1 m away shown in Figure 5c. The VCSEL was emitted at the wavelength of 940 nm, and the laser pulse was set to 1 ns. The algorithm was tested under a halogen lamp with a wide spectral range (350–2500 nm), and the background count was 240 kpcs on average in the indoor environment. To increase the robustness of the algorithm, the first stage needs to sample more data than the two later stages. As demonstrated in Figure 1a, the VCSEL was driven 2 k, 1 k, and 1 k times in the multi-scale histogram mode for each stage.

**Figure 6:**
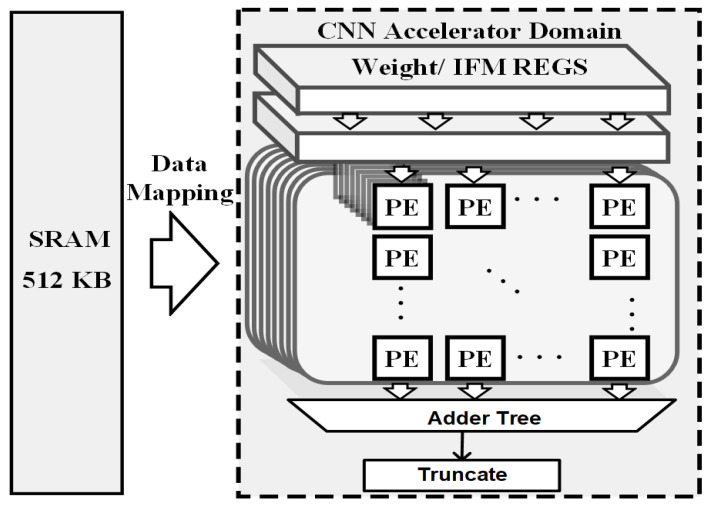
The adopted DLA architecture diagram.

To make the neural network inference time acceptable, a deep learning accelerator (DLA) is designed in this work in the algorithm end. As shown in Figure 6, the DLA is composed of 512 MACs and 512 KB on-chip ping-pong SRAM for storing the immediate features and weights. The process element (PE) array is configured as 16 atomic channels and 32 atomic kernel operations. The accelerating performance estimation for the proposed neural network was conducted by modeling the DLA with the deep learning accelerator tool in Pytorch. The adopted DLA was implemented using Verilog and synthesized at 500 MHz with a DC compiler under a TSMC 40 nm process. The synthesized system performance is shown in Table 1, and the power consumption and frame rate comparison with other LiDAR systems are reported in Table 2.

## 5. Results and Discussion

**Figure 7:**
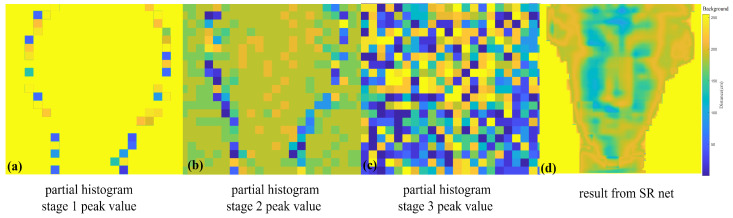
Test results in the lab: (**a**–**c**) Peak values of multi-scale histogram bins in 3 stages. (**d**) Result of target statue after application of SR-Net.

The validation results of the proposed neural network on hardware are shown in Figure 7. In Figure 7a–c, the peak maps of the multi-scale histogram coincide with the simulated data, which supports the feasibility of the algorithm on hardware. Regarding the edges of the detected objects, the receiver is triggered by some photons reflected by the background. In Figure 7a, the distance values on the outline are affected by background points, which show various colors, i.e., flying noise. According to the output result in Figure 7d, the proposed neural network was effective in extracting the depth map of this experimental set and recovered the details of the statue well.

**Figure 8:**
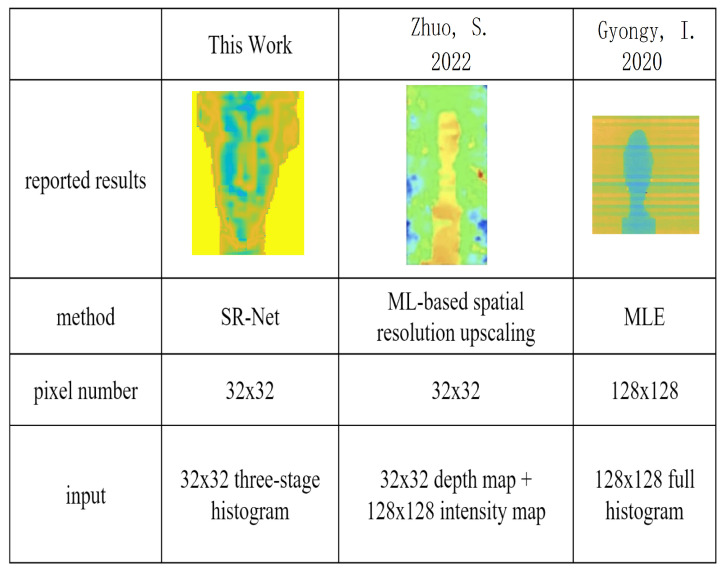
Performance comparison of this work with state-of-the-art dToF LiDAR systems [16] and classical dToF estimation method [10].

The performance of related research works is summarized in Figure 8. The SR-Net proposed in this work recovered more detailed depth features of the detected object. In Figure [16], the same backbone as SR-Net was adopted, but the ML-based spatial resolution up-scaling model proposed in [16] failed to improve the SNR, and spot noise was clear. In [10], the result kept the intrinsic distortion characteristics of SPAD sensors, including the line offsets, which can be corrected using a convolutional neural network.

**Figure 9:**
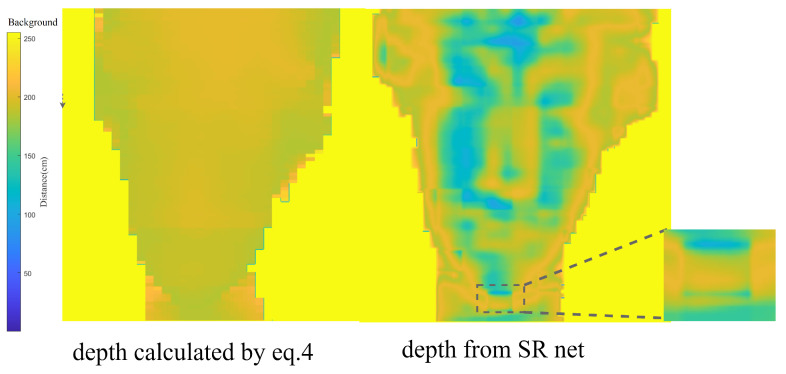
Comparison with maximum likelihood estimation. The “grid effect” generated by the up-sampling neural network is shown as a sub-image.

Figure 9 shows the different results obtained using maximum likelihood estimation (Equation (4)) and SR-Net. Compared with the 128 × 128 array output, more dedicated features of the statue can be observed, but less smooth. By zooming in on the details in the sub-figure, the common “grid effect” of the up-sampling network can be observed in this inference result. The grid effect can be eliminated by adding other information, for example, an RGB image, or by expanding the neural network size. The proposed neural network combined with the probabilistic encoder and SR-Net is a pragmatic method to use raw histogram bin data as the inputs to up-sample a high-resolution depth map. The proposed neural network combined with the probabilistic encoder and SR-Net is a practical method to employ raw histogram bin data as inputs to up-sample a high-resolution depth map.

## 6. Conclusions

In this work, compared with the on-chip histogram peak calculation algorithm, the probabilistic encoder and SR-Net combined neural network is proposed for up-sampling depth maps with multi-scale histogram outputs. Regarding the algorithm part, this work adopts the encoder network of a probabilistic encoder, which firstly saves hardware consumption for detecting the peak value of histograms in SPAD imaging systems. To verify the algorithm, a simulated dataset based on the SPAD characteristic was generated using the Middlebury dataset. By training the up-sampled network, the method proposed in this work recovered a 16×-size depth image with RMSE = 0.022 m on the generated dataset. Regarding the hardware platform, the multi-scale histogram bins from a 32 × 32 sensor imaging system are extracted and re-scaled up to a 128 × 128 depth map with rich details. By implanting the algorithm on a deep learning accelerator, the latency of the entire neural network was decreased, and the frame rate reached 20 fps, which is competitive with respect to other state-of-the-art works. The proposed SPAD imaging system offers a perspective on hardware and software co-design. Furthermore, this work could be capable of expanding up-sampling from 32× to 64× in the future.

## Figures and Tables

**Table 1:** Synthesis results of the DLA.

PE number	512
Frequency (MHz)	500
Area (mm^2^)	2.55
Power (mW)	306.82
Energy efficiency (TOPS/W)	2

**Table 2:** System performance summary and comparison table.

Parameter	This Work	[17]	[18]
Pixel array	**16 × 16**	80 × 60	48 × 40
Wavelength (nm)	**940**	905	905
TDC type	**In-Pixel**	In-Pixel	In-Pixel
TDC frequency (MHz)	**10**	0.1	0.1
TDC bit width (bits)	**Multi-stage**	Coarse 6	Coarse 7
	**12**	Fine 7	Fine 6
TDC resolution (ps)	**120**	100–5000	90–5000
Frame rate (fps)	**20**	30	None
Depth precision (cm)	**2**	1.5	2.34–4
Power (mW)	**150 (sensor)**	1500	840
	**306.82 (DLA)**		
Imaging resolution	**128 × 128**	80 × 60	42 × 25

## Data Availability

Data sharing not applicable.
